# Assessment of Temporal Patterns and Patient Factors Associated With Oseltamivir Administration in Children Hospitalized With Influenza, 2007-2020

**DOI:** 10.1001/jamanetworkopen.2022.33027

**Published:** 2022-09-23

**Authors:** Patrick S. Walsh, David Schnadower, Yin Zhang, Sriram Ramgopal, Samir S. Shah, Paria M. Wilson

**Affiliations:** 1 Department of Pediatrics, Section of Pediatric Emergency Medicine, Medical College of Wisconsin, Milwaukee; 2 Division of Emergency Medicine, Cincinnati Children’s Hospital Medical Center, Cincinnati, Ohio; 3 Department of Pediatrics, University of Cincinnati College of Medicine, Cincinnati, Ohio; 4 Division of Biostatistics and Epidemiology, Cincinnati Children’s Hospital Medical Center, Cincinnati, Ohio; 5 Ann and Robert H. Lurie Children’s Hospital of Chicago, Division of Emergency Medicine, Department of Pediatrics, Northwestern University Feinberg School of Medicine, Chicago, Illinois; 6 Division of Hospital Medicine, Cincinnati Children’s Hospital Medicine Center, Cincinnati, Ohio

## Abstract

**Importance:**

Oseltamivir therapy is recommended for all pediatric inpatients with influenza, particularly those with high-risk conditions, although data regarding its uptake and benefits are limited.

**Objective:**

To describe temporal patterns and independent patient factors associated with the use of oseltamivir and explore patterns in resource use and patient outcomes among children hospitalized with influenza.

**Design, Setting, and Participants:**

This multicenter retrospective cross-sectional study was conducted at 36 tertiary pediatric hospitals participating in the Pediatric Health Information System in the US. A total of 70 473 children younger than 18 years who were hospitalized with influenza between October 1, 2007, and March 31, 2020, were included.

**Exposures:**

Hospitalization with a diagnosis of influenza.

**Main Outcomes and Measures:**

The primary outcome was the use of oseltamivir, which was described by influenza season and by hospital. Patient factors associated with oseltamivir use were assessed using multivariable mixed-effects logistic regression models. Secondary outcomes were resource use (including antibiotic medications, chest radiography, supplemental oxygen, positive pressure ventilation, central venous catheter, and intensive care unit [ICU]) and patient outcomes (length of stay, late ICU transfer, 7-day hospital readmission, use of extracorporeal membrane oxygenation, and in-hospital mortality), which were described as percentages per influenza season.

**Results:**

Among 70 473 children hospitalized with influenza, the median (IQR) age was 3.65 (1.05-8.26) years; 30 750 patients (43.6%) were female, and 39 715 (56.4%) were male. Overall, 16 559 patients (23.5%) were Black, 36 184 (51.3%) were White, 14 133 (20.1%) were of other races (including 694 American Indian or Alaska Native [1.0%], 2216 Asian [3.0%], 372 Native Hawaiian or Pacific Islander [0.5%], and 10 850 other races [15.4%]), and 3597 (5.1%) were of unknown race. A total of 47 071 patients (66.8%) received oseltamivir, increasing from a low of 20.2% in the 2007-2008 influenza season to a high of 77.9% in the 2017-2018 season. Use by hospital ranged from 43.2% to 79.7% over the entire study period and from 56.5% to 90.1% in final influenza season studied (2019-2020). Factors associated with increased oseltamivir use included the presence of a complex chronic condition (odds ratio [OR], 1.42; 95% CI, 1.36-1.47), a history of asthma (OR, 1.31; 95% CI, 1.23-1.38), and early severe illness (OR, 1.19; 95% CI, 1.13-1.25). Children younger than 2 years (OR, 0.81; 95% CI, 0.77-0.85) and children aged 2 to 5 years (OR, 0.83; 95% CI, 0.79-0.88) had lower odds of receiving oseltamivir. From the beginning (2007-2008) to the end (2019-2020) of the study period, the use of antibiotic medications (from 74.4% to 60.1%) and chest radiography (from 59.2% to 51.7%) decreased, whereas the use of oxygen (from 33.6% to 29.3%), positive pressure ventilation (from 10.8% to 7.9%), and central venous catheters (from 2.5% to 1.0%) did not meaningfully change. Patient outcomes, including length of stay (median [IQR], 3 [2-5] days for all seasons), readmissions within 7 days (from 4.0% to 3.4%), use of extracorporeal membrane oxygenation (from 0.5% to 0.5%), and in-hospital mortality (from 1.1% to 0.8%), were stable from the beginning to the end of the study period.

**Conclusions and Relevance:**

In this cross-sectional study of children hospitalized with influenza, the use of oseltamivir increased over time, particularly among patients with high-risk conditions, but with wide institutional variation. Patient outcomes remained largely unchanged. Further work is needed to evaluate the impact of oseltamivir therapy in this population.

## Key Points

**Question:**

What are the temporal patterns in the use of oseltamivir among children hospitalized with influenza, and what patient factors are associated with its use?

**Findings:**

In this cross-sectional study of 70 473 children hospitalized with influenza in the US between 2007 and 2020, oseltamivir use increased over time, from 20.2% in the 2007-2008 influenza season to a peak of 77.9% in the 2017-2018 season, with wide institutional variation. Oseltamivir was used more frequently among children who were critically ill, children who were older, and children who had high-risk conditions.

**Meaning:**

This study’s findings suggest that although the use of oseltamivir has increased over time, further work is needed to address guideline adherence and evaluate the impact of oseltamivir administration for outcomes.

## Teaser

This cross-sectional study uses data from the Pediatric Health Information System to describe temporal patterns and patient factors associated with the use of oseltamivir therapy and examine patterns in resource use and patient outcomes among children hospitalized with influenza from 2007 to 2020.

## Introduction

Influenza is a seasonal illness with annual variability that imposes substantial burdens on individuals and the health care system. Influenza results in an estimated 2.8 to 10.7 million symptomatic illnesses, 1.6 to 6.1 million outpatient medical visits, 11 000 to 45 000 hospitalizations, and 110 to 600 excess deaths per year among children.^1^ Oseltamivir, which was first approved by the US Food and Drug Administration in 1999, remains the primary antiviral agent used to treat influenza. Since 2007 and 2009, respectively, guidelines from the American Academy of Pediatrics^2,3,4^ and the Infectious Diseases Society of America^5^ have recommended antiviral treatment with oseltamivir for all inpatients and outpatients with influenza who have high-risk conditions. Patients at high risk include those with complex chronic conditions, immunosuppression, or extreme obesity; those receiving long-term aspirin therapy; those of American Indian or Alaska Native race; those residing in chronic care facilities; and those younger than 5 years, especially patients younger than 2 years.^6,7^

It is unclear to what extent these guidelines have been adopted in pediatric settings and what factors are associated with oseltamivir use. Two multicenter studies of pediatric inpatients using data through 2015 found that only 69%^8^ and 72%^9^ received antiviral medications for influenza, with parenteral antiviral agents such as peramivir rarely used. Although 1 study^8^ reported a substantial increase (from 25% to 69%) in oseltamivir use in pediatric hospitals during the 2009 novel influenza A (H1N1) pandemic, with subsequent stability in the following seasons, the other study^9^ found steadily increasing rates of oseltamivir administration from 2010 to 2015. Data on the use of oseltamivir among patients with high-risk conditions and among those admitted to an intensive care unit (ICU) have been inconsistent.^8,9,10,11^ There is debate regarding whether the use of oseltamivir is associated with improved patient outcomes or decreased resource use among the inpatient population.^12^ The objectives of this cross-sectional study were therefore to describe temporal patterns and assess independent patient factors associated with the use of oseltamivir among children hospitalized with influenza between 2007 and 2020 and to explore patterns in patient outcomes and resource use over the same period.

## Methods

### Study Design

We performed a multicenter retrospective cross-sectional study of children with an inpatient discharge diagnosis of influenza using the Pediatric Health Information System (PHIS), an administrative database that contains inpatient, emergency department, ambulatory surgical procedures, and observation encounter–level data from more than 50 nonprofit tertiary care pediatric hospitals in the US.^13^ These hospitals are affiliated with the Children’s Hospital Association (Lenexa, Kansas). The quality and reliability of PHIS data are assured through a joint effort between the Children’s Hospital Association and participating hospitals. Data are deidentified at the time of data submission and are subject to a quality review before inclusion in the database.^13^ Patients in the PHIS are assigned an encrypted medical record number that can be used for longitudinal tracking of encounters over time. The study was approved by the institutional review board of Cincinnati Children’s Hospital Medical Center. Because all data were deidentified, the study was granted a waiver of informed consent. This study followed the Strengthening the Reporting of Observational Studies in Epidemiology (STROBE) reporting guideline for cross-sectional studies.

### Study Population

We included all inpatients younger than 18 years who were discharged from a participating hospital between October 1, 2007, and March 31, 2020, with a primary or secondary discharge diagnosis of influenza, defined as *International Classification of Diseases, Ninth Revision* (*ICD-9*), codes 487 or 488 or *International Statistical Classification of Diseases, Tenth Revision* (*ICD-10*), codes J09, J10, or J11.^14^ We used diagnostic codes for case definitions because primary clinical and laboratory data to confirm diagnoses are not available in the PHIS. Diagnostic codes have been previously used to identify pediatric patients hospitalized with laboratory-confirmed influenza with high specificity and positive predictive value.^15,16^

We excluded adult patients (aged ≥18 years) and encounters in which the patient was transferred to another hospital at discharge. We also excluded encounters from PHIS hospitals that did not report clinical and billing data for the entire study period.

### Measurements

We extracted patient characteristics, including age, sex, race, primary insurance, past diagnoses, and the presence of complex chronic conditions, from the PHIS database. In PHIS, race is collected per hospital-specific practices and is included as part of the administrative data for each encounter. We categorized age into 3 groups (<2 years, 2-5 years, and >5 years) in accordance with influenza guideline definitions of patients at high risk.^6^ We defined complex chronic conditions using a diagnostic code classification system reported by Feudtner et al,^17^ which captures chronic conditions likely to be severe enough to require hospitalization at a tertiary pediatric center. We defined history of asthma as the presence of any diagnosis of asthma (*ICD-9* code 493 or *ICD-10* code J45) in a previous encounter recorded within the PHIS.

We extracted information about treatment, which included the use of oseltamivir and other resources, such as antibiotic medications, supplemental oxygen, chest radiography, positive pressure ventilation, central venous catheters, and ICU care. Use of each resource was defined as having at least 1 calendar day with a billing charge for that resource during hospital admission. For antibiotic medications, we included oral, intravenous, and intramuscular anti-infective agents and did not include antiviral agents. We defined positive pressure ventilation as a billing charge for high-flow nasal cannula, continuous positive airway pressure, bilevel positive airway pressure, or mechanical ventilation. We extracted information about patient outcomes, including length of stay (LOS), late ICU transfer, 7-day hospital readmission, use of extracorporeal membrane oxygenation (ECMO), and in-hospital mortality. Length of stay was calculated as the number of calendar days between the admission date and the discharge date. Late ICU transfer was defined as transfer to the ICU on hospital day 2 or later after being admitted to a general ward. In-hospital mortality and ECMO use were identified using corresponding signals in the PHIS data. For the assessment of factors associated with oseltamivir use, we limited the analysis of ICU use and the need for a central venous catheter or positive pressure ventilation to early in the hospitalization, which we categorized as a billing charge on hospital day 0 or day 1. We limited the use of these therapies to day 0 or day 1 of hospitalization so the analysis would best capture the severity of illness at presentation.

### Outcome

Our primary outcome was the use of oseltamivir, which was defined as a billing charge for at least 1 day of oseltamivir therapy during hospital admission. Secondary outcomes were resource use (antibiotic medications, chest radiography, supplemental oxygen, positive pressure ventilation, central venous catheter, and ICU care) and patient outcomes (LOS, ICU admission, late ICU transfer, 7-day hospital readmission, ECMO use, and in-hospital mortality). For resource use outcomes, we chose common therapies used for the treatment of children with infectious or cardiorespiratory symptoms associated with influenza infection.

### Statistical Analysis

We described patient characteristics of those who did and did not receive oseltamivir using frequencies with proportions to report categorical variables and medians with IQRs to report continuous variables. We calculated the proportion of patients who received oseltamivir by institution, US Census region, and influenza season. We defined influenza season as starting on July 1 of each year, consistent with previous epidemiological studies of influenza.^18^ We described temporal patterns as well as institutional and geographic variations in the use of oseltamivir using medians with IQRs. We also described patterns in oseltamivir use among patients by high-risk subgroup. We described patterns in resource use and patient outcomes by calculating percentages for each influenza season and generating plots. The unit of analysis was the individual patient encounter.

To assess independent patient factors associated with oseltamivir use, we used generalized linear mixed-effects models with patient factors as fixed effects and hospital and influenza season as random effects to account for clustering and seasonality. The dependent variable was the binary outcome of oseltamivir use. Variables included in the models as fixed effects were determined a priori according to biological plausibility and included demographic characteristics, the presence of a high-risk condition (including younger age groups, a history of asthma, and the presence of a complex chronic condition), and the presence of severe illness at presentation (defined as ICU admission or a composite variable of the need for positive pressure ventilation or a central venous catheter). We evaluated all variables in univariate and multivariable models and calculated odds ratios (ORs) for the use of oseltamivir for each variable. For the multivariable model, we calculated Tukey-adjusted CIs to avoid multiple comparison problems. Statistical analyses were conducted using R software, version 4.0.2 (R Foundation for Statistical Computing), and SAS software, version 9.4 (SAS Institute Inc). The threshold for statistical significance was 2-sided *P* < .05.

## Results

### Study Population

Over the 13-year study period, we identified 70 926 children with inpatient hospitalizations for influenza that met our inclusion criteria. We excluded 453 patients who were transferred to another hospital at discharge, leaving 70 473 children with inpatient hospitalizations at 36 tertiary pediatric hospitals included in the cohort. The median (IQR) age of the cohort was 3.65 (1.05-8.26) years; 30 750 patients (43.6%) were female, and 39 715 (56.4%) were male. In total, 16 559 patients (23.5%) were Black, 36 184 (51.3%) were White, 14 133 (20.1%) were of other races (including 694 American Indian or Alaska Native [1.0%], 2216 Asian [3.0%], 372 Native Hawaiian or Pacific Islander [0.5%], and 10 850 other races [15.4%]), and 3597 (5.1%) were of unknown race. Over the study period, 47 071 patients (66.8%) received oseltamivir, and 23 402 children (33.2%) did not. Demographic and clinical characteristics of children who did and did not receive oseltamivir are shown in Table 1.

**Table 1.** Demographic and Clinical Characteristics of Children Hospitalized With Influenza, 2007-2020

Characteristic	Patients, No. (%)	
	Received oseltamivir (n = 47 071)	Did not receive oseltamivir (n = 23 402)
Age, y		
Median (IQR)	4.08 (1.22-8.77)	2.86 (0.80-7.15)
Category		
<2	16 252 (34.5)	9891 (42.3)
2-5	9782 (20.8)	4913 (21.0)
>5	21 037 (44.7)	8598 (36.7)
Sex		
Female	20 476 (43.5)	10 274 (43.9)
Male	26 591 (56.5)	13 124 (56.1)
Race		
Black	11 557 (24.6)	5002 (21.4)
White	23 275 (49.4)	12 909 (55.2)
Other^a^	9970 (21.2)	4163 (17.8)
Unknown	2269 (4.8)	1328 (5.7)
Insurance type		
Public	30 045 (63.8)	13 992 (59.8)
Private	14 624 (31.1)	7960 (34.0)
Other	1561 (3.3)	1071 (4.6)
Unknown	841 (1.8)	379 (1.6)
Region		
Midwest	11 165 (23.7)	5519 (23.6)
Northeast	4549 (9.7)	2502 (10.7)
South	17 529 (37.2)	9783 (41.8)
West	13 828 (29.4)	5598 (23.9)
Complex chronic condition	22 195 (47.2)	9232 (39.4)
History of asthma^b^	5876 (12.5)	2490 (10.6)
Early ICU care^c^	11 614 (24.7)	4638 (19.8)

Abbreviation: ICU, intensive care unit.

^a^ Other races include American Indian or Alaska Native (694 patients [1.0%]), Asian (2216 patients [3.0%]), Native Hawaiian or Pacific Islander (372 patients [0.5%]), and other (10 850 patients [15.4%]).

^b^ History of asthma does not include patients with complex chronic conditions.

^c^ Defined as receipt of care in an ICU on hospital day 0 or hospital day 1.

### Patterns and Variation in Oseltamivir Use

**Figure 1.**
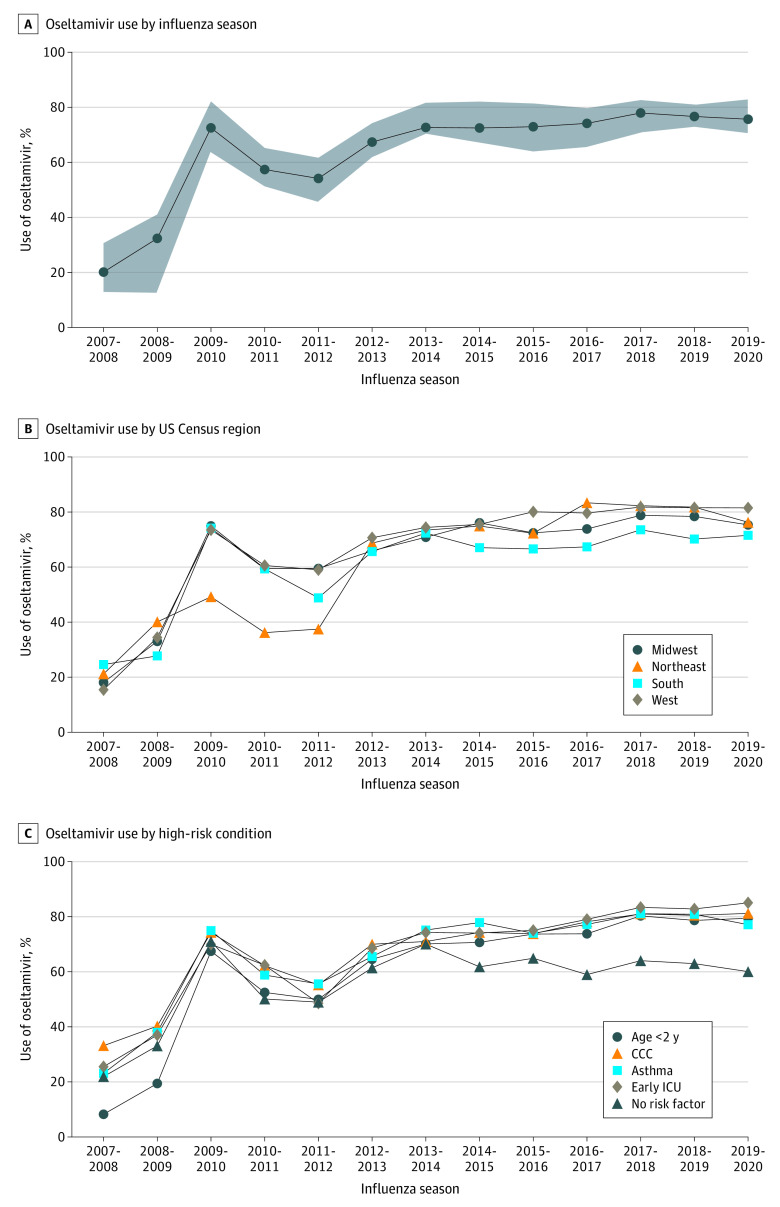
Oseltamivir Use Among Children Hospitalized With Influenza in US Children’s Hospitals, 2007-2020 A, Shaded area represents IQRs. CCC indicates complex chronic condition; and ICU, intensive care unit.

Oseltamivir use increased over the study period, starting at 20.2% in the 2007-2008 influenza season and reaching a maximum of 77.9% in the 2017-2018 season (with 22.1% of children not receiving oseltamivir in 2017-2018) (Figure 1). Institutional practice varied, with oseltamivir use ranging from 43.2% to 79.7% by hospital for the overall study period (median [IQR], 66.3% [62.8%-69.2%]). In the 2019-2020 season, oseltamivir use by hospital ranged from 56.5% to 90.1%. Geographic regions generally followed the overall pattern, differing by 3% to 25% depending on the year, with overall proportions of 66.9% in the Midwest, 64.5% in the Northeast, 64.2% in the South, and 71.1% in the West.

Within high-risk subgroups, oseltamivir administration was higher among patients who received treatment in the ICU vs those who did not (71.0% vs 65.4%), among patients with a complex chronic condition vs no complex chronic condition (70.6% vs 63.7%), and among patients with a history of asthma vs no history of asthma (70.2% vs 66.3%); however, oseltamivir use was lower among younger patients (62.2% for children aged <2 years and 66.5% for children aged 2-5 years) vs older patients (71.6% for children aged >5 years) (Figure 1). Among younger patients, oseltamivir use was highest in 2013-2014 (69.9%), decreasing in the latter study period (59.9% in 2019-2020), particularly among infants (only 8.4% received oseltamivir before 2009). Detailed patterns within age groups are shown in the eFigure in the Supplement.

Patients without high-risk factors had lower oseltamivir use than those with high-risk factors starting in 2014 (use among those without high risk peaked at 69.8% in the 2013-2014 season and decreased to 59.8% in the 2019-2020 season). Because oseltamivir is an enteral medication, we also evaluated peramivir use, finding that 264 children (0.3%) received peramivir; of those, 182 children received oseltamivir, and 82 did not.

### Factors Associated With Oseltamivir Use

The results of the univariate and multivariable generalized linear mixed-effects models are shown in Table 2. Patient factors independently associated with higher oseltamivir administration in our multivariable model included Black race (OR, 1.28; 95% CI, 1.21-1.36), public insurance (OR, 1.17; 95% CI, 1.12-1.24), the presence of a complex chronic condition (OR, 1.42; 95% CI, 1.36-1.47), a history of asthma (OR, 1.31; 95% CI, 1.23-1.38), early ICU care (OR, 1.19; 95% CI, 1.13-1.25), and the need for early positive pressure ventilation or a central venous catheter (OR, 1.21; 95% CI, 1.14-1.29). Children younger than 2 years (OR, 0.81; 95% CI, 0.77-0.85) and children aged 2 to 5 years (OR, 0.83; 95% CI, 0.79-0.88) had lower odds of receiving oseltamivir when accounting for influenza season and hospital as random effects.

**Table 2.** Factors Associated With Oseltamivir Use Among Children Hospitalized With Influenza

Characteristic	Patients, No. (%)	OR (95% CI)	
		Univariate	Multivariable
Age	70 473 (100)	1.04 (1.04-1.04)	NA
Age category, y			
<2	26 143 (37.1)	0.67 (0.64-0.69)	0.81 (0.77-0.85)
2-5	14 695 (20.9)	0.81 (0.78-0.85)	0.83 (0.79-0.88)
>5	29 635 (42.1)	1 [Reference]	1 [Reference]
Race			
Black	16 559 (23.5)	1.30 (1.25-1.36)	1.28 (1.21-1.36)
White	36 184 (51.3)	1 [Reference]	1 [Reference]
Other^a^	14 133 (20.1)	1.23 (1.17-1.29)	1.20 (1.12-1.28)
Unknown	3597 (5.1)	1.08 (1.00-1.16)	1.07 (0.96-1.20)
Insurance type			
Private	22 584 (32.0)	1 [Reference]	1 [Reference]
Public	44 037 (62.5)	1.16 (1.12-1.20)	1.17 (1.12-1.24)
Other	2632 (3.7)	0.80 (0.73-0.87)	1.15 (1.02-1.30)
Unknown	1220 (1.7)	1.21 (1.06-1.38)	1.69 (1.39-2.05)
Medical history			
Complex chronic condition	31 427 (44.6)	1.40 (1.36-1.45)	1.42 (1.36-1.47)
Asthma^b^	8366 (11.9)	1.20 (1.14-1.27)	1.31 (1.23-1.38)
Severe illness			
Early ICU care^c^	16 252 (23.1)	1.36 (1.31-1.42)	1.19 (1.13-1.25)
Need for positive pressure ventilation or central venous catheter	9539 (13.5)	1.49 (1.42-1.57)	1.21 (1.14-1.29)

Abbreviations: ICU, intensive care unit; NA, not applicable; OR, odds ratio.

^a^ Other races include American Indian or Alaska Native (694 patients [1.0%]), Asian (2216 patients [3.0%]), Native Hawaiian or Pacific Islander (372 patients [0.5%]), and other (10 850 patients [15.4%]).

^b^ Does not include patients with a complex chronic condition.

^c^ Defined as receipt of care in an ICU on hospital day 0 or hospital day 1.

### Patterns in Resource Use and Patient Outcomes

**Figure 2.**
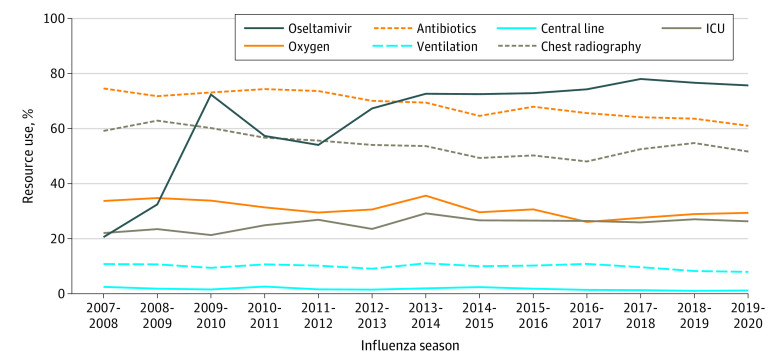
Resource Use Among Children Hospitalized With Influenza in US Children’s Hospitals, 2007-2020 ICU indicates intensive care unit.

Among all patients included in the study, 67.8% received antibiotic medications, 30.6% required supplemental oxygen, 9.6% required ventilation (continuous positive airway pressure, bilevel positive airway pressure, or mechanical ventilation), 1.6% received a central venous catheter, and 54.3% received chest radiography. Patterns in resource use by year are shown in Figure 2. From the beginning (2007-2008) to the end (2019-2020) of the study period, decreasing use of antibiotic medications (from 74.4% to 60.1%) and chest radiography (from 59.2% to 51.7%) was observed, whereas the use of oxygen (from 33.6% to 29.3%), central venous catheters (from 2.5% to 1.0%), and positive pressure ventilation (from 10.8% to 7.9%) was stable over time. The median (IQR) LOS was 3 (2-5) days. A total of 631 patients (0.9%) died, 397 (0.6%) received ECMO, 2794 (4.0%) had a readmission within 7 days, 17 630 (25.0%) required ICU care, and 1544 (2.2%) required transfer to ICU care after hospital day 1.

**Figure 3.**
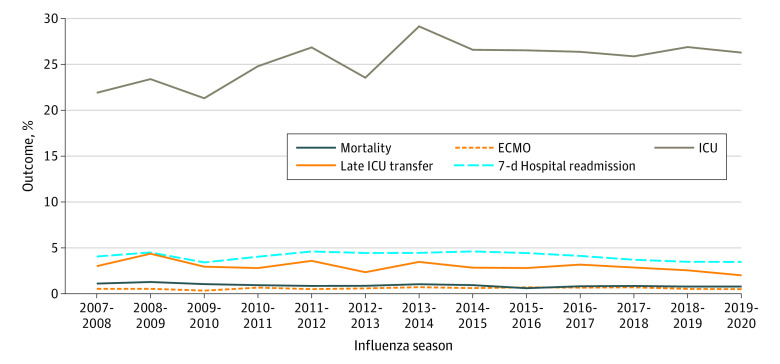
Outcomes Among Children Hospitalized With Influenza in US Children’s Hospitals, 2007-2020 ECMO indicates extracorporeal membrane oxygenation; and ICU, intensive care unit.

Patterns in patient outcomes by year are shown in Figure 3. The use of ICU care increased slightly over time, from 21.8% in the 2007-2008 season to 26.3% in the 2019-2020 season, whereas LOS (median [IQR], 3 [2-5] days for all seasons), ECMO use (from 0.5% to 0.5%), in-hospital mortality (from 1.1% to 0.8%), 7-day readmissions (from 4.0% to 3.4%), and late ICU transfers (from 3.0% to 2.0%) were stable from the beginning to the end of the study period.

## Discussion

In this multicenter cross-sectional study of children hospitalized with influenza, we found that oseltamivir use increased over time. Despite this increase, 22.1% of children still did not receive oseltamivir during hospitalization in the highest-use season. Although patients with certain high-risk conditions (such as the presence of a complex chronic condition or a history of asthma) and patients requiring ICU care or invasive procedures early in their hospitalization were more likely to receive oseltamivir, this pattern was not found among younger patients. The use of antibiotic medications and chest radiography decreased over time, whereas the use of oxygen, central venous catheters, and positive pressure ventilation remained stable. Patient outcomes, including LOS, in-hospital mortality, late ICU transfers, and hospital readmissions, did not change during the study period.

Our finding that 66.8% of children hospitalized with influenza received oseltamivir is consistent with previous studies of US children’s hospitals, which reported that 69%^8^ and 72%^9^ of children received antiviral medications before 2015, with substantial institutional variation (42%-90% range of use between hospitals in the 2014-2015 season).^8^ The rate of oseltamivir use in our study was higher than that the rate found in a recent study of Canadian children’s hospitals,^19^ which reported that only 41% of patients received treatment with antiviral agents from 2010 to 2019, although the researchers noted an increasing proportion each year, peaking at 59% in the 2018-2019 season. Consistent with our results, the Canadian study^19^ found wide institutional variation in use and increased odds of receiving antiviral therapy among children with chronic conditions, children who received treatment in the ICU or required respiratory support, and older children. Using population-based surveillance data, Appiah et al^9^ also found steadily increasing use from 2010 to 2015 in the US, higher use among patients with high-risk conditions, and a large increase in use among young children around the time of US Food and Drug Administration approval of oseltamivir to include children younger than 1 year in 2012.

The characteristics of patients hospitalized with influenza and the resource use observed in this study were similar to those reported in previous literature. Consistent with a previous study describing influenza in children^20^ but contrary to data from a study of adults with influenza,^14^ we found a male predominance in the cohort. We observed decreasing use of antibiotic medications, which may be associated with more readily available influenza testing, resulting in more targeted therapy or an overall reduction in antibiotic use by PHIS hospitals. Previous studies reported that 60% of all inpatients received antibiotic medications in 2008,^21^ whereas 36% of inpatients received an antibiotic medication in 2017.^22^ The use of chest radiography has also been reported to be decreasing among children with respiratory illness,^23^ which was consistent with our results. Notably, although previous work has revealed increasing use of ECMO in adults with influenza,^24^ we did not find increases in ECMO use among children. Future work might evaluate the impact of resource use variation for costs.

In the years since 2015, the overall use of oseltamivir has remained stable. Although groups with high-risk conditions continued to have increased use, patients without an identified high-risk factor began to have decreasing use (peak of 69.8% in 2013-2014 that decreased to 59.8% in 2019-2020). Jefferson et al^25^ published a systematic review in 2014 that raised substantial concerns about the quality of evidence for the benefits of oseltamivir, which eventually resulted in the World Health Organization downgrading oseltamivir from a core drug to a complementary drug in 2017.^26^ Thus, although practice variation and deviation from clinical guidelines are common in medical practice,^27,28^ in the case of oseltamivir, variation and deviation may be associated with uncertainties regarding its efficacy.^25,29,30^

The patterns in patient outcomes found in this study did not dispel these uncertainties. Although previous observational studies found small improvements in LOS,^31,32^ readmission,^33^ and mortality^34^ among patients receiving oseltamivir, our results did not corroborate those findings. Our data suggested a large increase in oseltamivir use early in the study period but no meaningful reductions in patient outcomes, such as LOS, 7-day readmissions, ECMO use, or in-hospital mortality. Although the data were raw and observational and did not account for severity of illness, changes in pathogen virulence, or changes in practice that could be associated with outcomes over time, these patterns suggest that further examination of the benefits of oseltamivir therapy in this population is warranted.

### Limitations

This study has several limitations. The PHIS is an administrative data set and does not contain clinical information, such as physical examination findings or laboratory test results; thus, we cannot confirm that all included patients had laboratory-confirmed influenza. Although administrative diagnostic codes have variable accuracy,^35^ influenza *ICD* codes have had good specificity and positive predictive value for laboratory-confirmed influenza.^15,16^ Furthermore, we do not have clinical data pertaining to how long patients had been ill before hospitalization, which could have implications for our findings because recommendations for oseltamivir therapy are strongest for patients who receive treatment early in the disease course.^7^

In our analyses of high-risk conditions, we did not include obesity because this condition has been reported to be inaccurately coded in inpatient settings.^36^ We also did not include patients of American Indian or Alaska Native race in the analyses as a separate racial category because numbers were small. Future data collection may seek to identify American Indian and Alaska Native patients to allow for the study of disparities, particularly because this group is known to be at high risk of complications associated with influenza.^6,7^ When categorizing patients with a history of asthma, there may have been varying lead times for previous encounters with an asthma diagnosis (ie, some patients had more time before the included encounter to meet the criteria than others) based on patient age, year, and date at which the hospital joined the PHIS, so it is possible some patients were misclassified.

We do not have access to outpatient visits in the PHIS, so we are unable to assess whether patients may have received partial or full courses of oseltamivir therapy as outpatients. We also do not have access to encounters outside of PHIS hospitals, so the true hospital readmission rate may be higher than reported. However, another study^37^ reported that relatively few pediatric patients have subsequent encounters at a different hospital.

## Conclusions

In this cross-sectional study, oseltamivir use among children hospitalized with influenza increased over time; however, this increase was only marginal over the last 5 years of the study period. In the highest-use season, 22.1% of children hospitalized with influenza did not receive oseltamivir despite guidelines recommending its use. Children who were critically ill, older children, and children with high-risk medical histories were more likely to receive oseltamivir. Patient outcomes remained unchanged despite higher use of oseltamivir, which suggests a closer examination of the benefits of oseltamivir therapy is warranted in this population.

## Notes

Supplement. **eFigure.** Patterns in Oseltamivir Use Within Pediatric Age Groups
