# Supplementary material for: Assessment of Temporal Patterns and Patient Factors Associated With Oseltamivir Administration in Children Hospitalized With Influenza, 2007-2020
Source: JAMA Netw Open. 2022 Sep 23;5(9):e2233027. doi: 10.1001/jamanetworkopen.2022.33027 (PMC9508650; doi:10.1001/jamanetworkopen.2022.33027)
Supplement: Supplement. — eFigure. Patterns in Oseltamivir Use Within Pediatric Age Groups [file jamanetwopen-e2233027-s001.pdf]

Supplementary Online Content

Walsh PS, Schnadower D, Zhang Y, Ramgopal S, Shah SS, Wilson PM. Assessment of temporal patterns and patient factors associated with oseltamivir administration in children hospitalized with influenza, 2007-2020. *JAMA Netw Open*. 2022;5(9):e2233027. doi:10.1001/jamanetworkopen.2022.33027

eFigure. Patterns in Oseltamivir Use Within Pediatric Age Groups

This supplementary material has been provided by the authors to give readers additional information about their work.

© 2022 Walsh PS et al. *JAMA Network Open*.

eFigure. Patterns in Oseltamivir Use Within Pediatric Age Groups

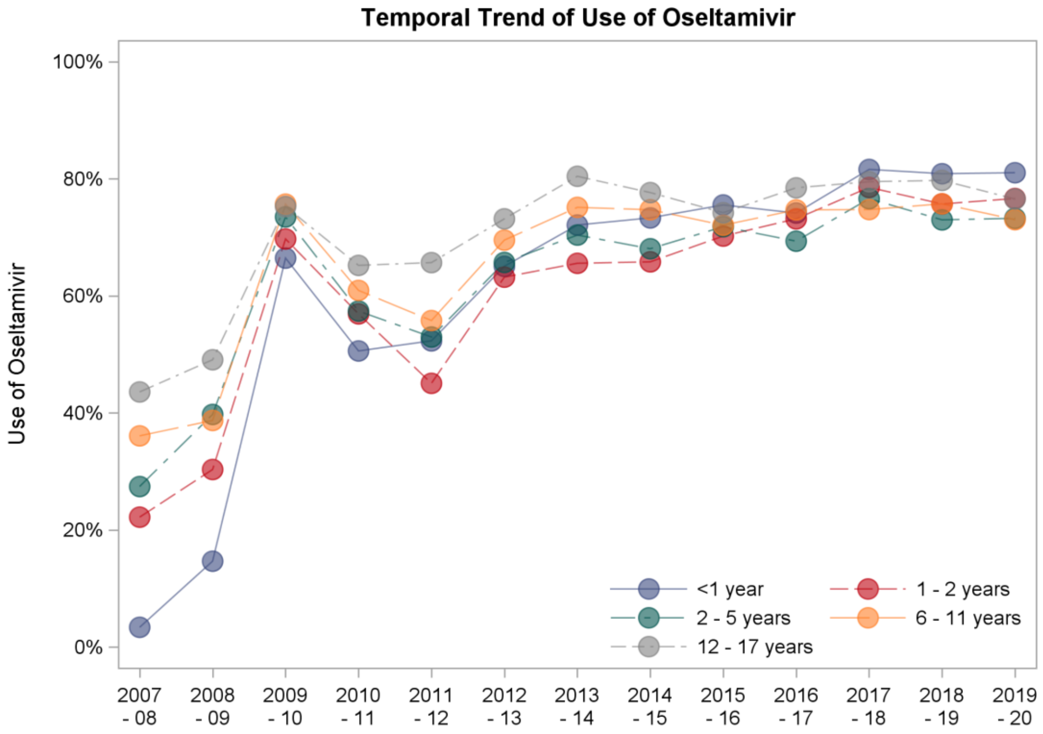

Oseltamivir use was very low in infants < 1 year of age prior to the emergency use authorization (EUA) by the US Food and Drug Administration in 2009.

© 2022 Walsh PS et al. *JAMA Network Open*.
